# Local Irradiation Sensitized Tumors to Adoptive T Cell Therapy via Enhancing the Cross-Priming, Homing, and Cytotoxicity of Antigen-Specific CD8 T Cells

**DOI:** 10.3389/fimmu.2019.02857

**Published:** 2019-12-11

**Authors:** Jin-Zhi Lai, Yan-Yang Zhu, Mei Ruan, Ling Chen, Qiu-Yu Zhang

**Affiliations:** ^1^Department of Basic and Clinical Research, Institute of Immunotherapy, Fujian Medical University, Fuzhou, China; ^2^Department of Oncology, Fujian Medical University Union Hospital, Fuzhou, China

**Keywords:** irradiation, adoptive T cell therapy, tumor infiltrating lymphocyte, cross-priming, chemokine

## Abstract

The successful generation of T cell-mediated immunity for the treatment of cancer has been a major focal point of research. One of the critical strategies of cancer immunotherapy is to efficiently activate antigen-specific CD8 T cells in the immunosuppressive tumor environment. Here, we used transgenic OT-I/CD45.2/Rag^−/−^ mice as a source of effector CD8 T cells to determine whether irradiation combined with adoptive T cell transfer therapy could improve T cell proliferation and effector function in murine tumor models. Local irradiation combined with adoptive T cell therapy showed a synergistic effect on tumor growth inhibition in mice. Mechanistically, irradiation increased the release of tumor-associated antigens, which facilitated cross-presentation of tumor-associated antigens by dendritic cells and the priming of antigen-specific T lymphocytes. Additionally, irradiation enhanced the homing of the antigen-specific T cells to tumor tissues via the increased release of CCL5, CXCL9, and CXCL11 from tumor cells. Moreover, irradiation enhanced the proliferation and effector function of both adoptively transferred T cells and endogenous antigen-specific T cells. Our findings provide evidence to support that local irradiation enhanced the therapeutic efficacy of adoptive T cell therapy for cancer, indicating that the combination of radiotherapy and adoptive T cell therapy may be a promising strategy for tumor treatment.

## Introduction

Radiotherapy, also known as irradiation, represents the standard-of-care treatment for localized cancer, and a palliative treatment for patients with widespread metastatic cancers (1). Overall, ~60% of patients with cancer received radiotherapy during the course of their disease (2). Irradiation was originally used because of ability to induce DNA damage, resulting in cell death via mitotic catastrophe, apoptosis, necrosis, and autophagy (3, 4). In addition to the direct cytotoxic effects, increasing evidence has demonstrated that irradiation could induce immunogenic cell death by involving the recruitment of the host's immune cells as a contributor to cancer treatment (5). Irradiation induced the release of tumor-associated antigens (TAAs) and danger-associated molecular patterns (DAMPs), promoting dendritic cell (DC) activation and cross-priming of naïve T cells (6). Furthermore, irradiation can stimulate the secretion of various chemokines, such as CXCL10 and CXCL16, which can attract effector T cells into the tumor microenvironment (7). However, radiotherapy alone is sometimes unable to completely eradicate advanced tumors. Thus, there has been considerable interest in combining radiotherapy with novel immunotherapies to increase the clinical benefit in cancer patients.

Adoptive T cell therapy is one of the adoptive cell therapy (ACT) options, including ACT with tumor-infiltrating lymphocytes (TILs), chimeric antigen receptor T cells (CAR-T), and engineered T cell receptor T cells (TCR-T) (8). The first study of adoptive T cell therapy was performed by Rosenberg et al., and the results showed that TILs can be effective against metastatic melanoma (9). Until now, adoptive T cell therapy has been conducted in a variety of tumor types, including renal cell cancer (10), cervical cancer (11), and non-small cell lung cancer (12). Although promising results have been shown in some clinical trials, adoptive T cell therapy still faces some challenges in treating solid tumors. The pathological barrier of the tumor microenvironment prevents immune cells from penetrating into tumors, which restrains the direct contact of adoptively transferred T cells with tumor cells and limits the efficacy of adoptive T cell therapy (13). Strategies to improve the viability and proliferation of adoptively transferred immune cells in the immunosuppressive tumor microenvironment have been a matter of intense debate.

A large number of new strategies have been used in preclinical animal models to enhance the trafficking of adoptively transferred immune cells to tumor tissues (14–16). Irradiation promotes the release of DAMPs and cytokines that are responsible for recruiting immune cells into the tumor tissues (2), providing a mechanistic rationale for combining irradiation with adoptive T cell therapy for cancer treatment. In this study, we sought to determine whether irradiation combined with adoptive T cell transfer therapy could improve T cell survival and function, and if so, to identify the immunological basis of this enhanced functionality. We found that irradiation increased the cross-presentation of TAAs and the proliferation of antigen-specific T lymphocytes. Our results revealed that irradiation can stimulate the secretion of various chemokines, which can attract effector T cells into the tumor microenvironment. Thus, local irradiation enhanced the effect of subsequent adoptive T cell transfer in mouse tumor models. Overall, these findings have important implications for combined treatment with irradiation and adoptive T cell therapy in the clinic.

## Materials and Methods

### Mice Strains and Cell Lines

Female C57BL/6 mice aged 5–6 weeks were purchased from the Experimental Animal Center of Fujian Medical University (Fuzhou, China). Recipient CD45.1 mice and donor OT-I/CD45.2/Rag^−/−^ mice were bred in-house. The donor OT-I/CD45.2/Rag^−/−^ mice express a transgenic T cell receptor (TCR) that recognizes the H-2Kb-restricted class I epitope of ovalbumin (OVA_257−264_, SIINFEKL). All mice were maintained in pathogen-free facilities at Fujian Medical University.

MC38-OVA and MC38 colorectal carcinoma cells were obtained from the laboratory of Dr. Lie-Ping Chen (Yale University). EG7-OVA and EL4 lymphoma cells were purchased from ATCC (Manassas, USA). All tumor cell lines were tested before use and found to be free of mycoplasma by PCR mycoplasma test kit (HuaAn Biotechnologies, China).

### Peptides and Antibodies

OVA_257−264_ (SIINFEKL, H-2Kb) peptides were synthesized by Life Technologies (Thermo Fisher Scientific, USA). The following anti-mouse antibodies used for analysis were purchased from eBioscience: CD3 (clone 17A2), CD4 (clone RM4-5), CD8 (clone 53-6.7), CD45.2 (clone 104), Vβ5.1 (clone MR9-4), IFN-γ (clone XMG1.2), TNF-α (clone MP6-XT22), CD11c (clone N418), SIINFEKL/H-2Kb (25-D1.16), IgG1 kappa isotype control (clone P3.6.2.8.1), IgG2a kappa isotype control (clone eBM2a), and Fixable Viability Dye eFluor 506. The following anti-mouse antibodies were purchased from BD Biosciences: CD45 (clone 30-F11), CD45.1 (clone A20), CD4 (clone GK1.5), CD3e (clone 145-2C11), CD8a (clone 53-6.7), and Fas (15A7). Samples were analyzed on a BD FACSVerse flow cytometer and analyzed using FlowJo software version 10 (BD Biosciences, USA).

### Phagocytosis Assay

Bone marrow-derived dendritic cells (BM-DCs) were prepared for the phagocytosis assay. Bone marrow cells were isolated from the femurs of mice and red blood cells were lysed following treatment with ammonium chloride potassium lysis buffer at room temperature for 3 min. Cells at a concentration of 1 × 10^6^ cells/ml were cultured in complete medium composed of RPMI-1640 medium supplemented with recombinant mouse IL-4 (10 ng/ml) and recombinant mouse GM-CSF (50 ng/ml) (PeproTech, USA) for 7 days, and then the non-adherent cells (BM-DCs) were harvested for co-culture with irradiated tumor cells.

MC38-OVA and EG7-OVA cells were subjected to 5 or 10 Gy of radiation (or sham-irradiation). The irradiated cells were incubated for 48 h in complete medium and then stained with 3 μM 5,6-carboxyfluorescein diacetate succinimidyl ester (CFSE) (Sigma-Aldrich, USA). BM-DCs were harvested and stained with anti-mouse CD11c at 4°C for 30 min, followed by co-culture with CFSE-labeled MC38-OVA or EG7-OVA in a 96-well-plate for 4 h. The phagocytosis of irradiated tumor cells was analyzed by flow cytometry. For microscopy analysis of cell engulfment, DCs and tumor cells that were cultured and stained as described above were mounted on a coverslip and assessed with a fluorescence microscope (Life Technologies, USA) at a magnification of 200×.

### Apoptosis Assay

MC38-OVA and EG7-OVA cells were subjected to 5 or 10 Gy of radiation (or sham-irradiation). After 48 h of incubation in complete medium, the irradiated cells were stained with Annexin V- PE and 7-AAD (eBioscience, USA) according to the manufacturer's instructions. The percentages of apoptotic tumor cells were quantified by flow cytometry. Late apoptotic cells were defined as annexin V and 7-AAD double-positive; early apoptotic cells were defined as annexin V positive but 7-AAD negative.

### T Cell Proliferation Assay

Irradiated or non-irradiated EG7-OVA cells were incubated with DCs for 4 h. After incubation, the DCs were magnetically isolated from splenocytes using the EasySep™ Mouse Pan-DC Enrichment Kit (STEMCELL Technologies, Canada) and co-cultured with CFSE-labeled naïve OT-I T cells for 72 h. Cell proliferation was analyzed by flow cytometry.

### T Cell Adoptive Transfer Experiments

Spleens were harvested from OT-I/CD45.2/Rag^−/−^ mice. OT-I T cells were isolated by negative selection (STEMCELL Technologies, Canada) and then labeled with CFSE according to the manufacturer's protocol. CFSE-labeled OT-I T cells were resuspended in 200 μl of PBS and transferred by intraperitoneal injection (5 × 10^6^ cells/mouse) into MC38-OVA tumor-bearing mice the day after local irradiation. Five days after irradiation, the mice were sacrificed, and the tumor and draining lymph node (DLN) suspensions were analyzed via flow cytometry. For the EG7-OVA model, donor OT-I T cells were obtained from OT-I/CD45.2/Rag^−/−^ mice, whereas the recipient mice were CD45.1 mice, thereby permitting *in vivo* isolation via the identification of cells with the congenic marker.

### Fluorescent Labeling of OT-I T Cells and Fluorescence Live Imaging (FLI)

DiR (PerkinElmer, USA) is a lipophilic near-infrared fluorescent cyanine dye (absorption/emission: 748/780 nm) used for labeling the cytoplasmic membrane. OT-I T cells were stained with DiR working solution (320 μg/ml) for 30 min at 37°C. DiR-labeled OT-I T cells were washed twice with PBS and then transferred by intraperitoneal injection (5 × 10^6^ cells/mouse) into MC38-OVA tumor-bearing mice. After the adoptive transfer of labeled OT-I T cells, mice were anesthetized with isoflurane (RWD Life Science Inc., Canada) and FLI was performed using the Xenogen IVIS-Spectrum Imaging System (Caliper Life Sciences Inc., USA) from day 1 to day 21. Living Image v.5.0 software (PerkinElmer, USA) was used to draw and calculate the regions of interest.

### Real-Time Quantitative PCR (RT-qPCR)

Tumor cells received 5 or 10 Gy of radiation (or sham-irradiation). After incubation in complete medium for 24 h, all cells were collected for RNA isolation. Total RNA was reverse-transcribed into cDNA using a Transcriptor First Strand cDNA Synthesis Kit (Roche, Germany) according to the manufacturer's instructions. Quantitative real-time PCR (qRT-PCR) was performed using a SYBR^®^ Prime Script™ RT-PCR Kit (Invitrogen, USA). The primers that were used are listed as follows: mCCL5 forward (5′-ACTGCATCTGCCCTAAGGTCTT-3′) and reverse (5′-TGCTTGAGGTGGTTGTGGAA-3′), mCXCL9 forward (5′-GTCCGCTGTTCTTTTCCTCTTG-3′) and reverse (5′-GGTGCTGATGCAGGAGCAT-3′), mCXCL10 forward (5′-GACCAGTAAGAAGATCCCCAACA-3′) and reverse (5′-GCCCAACCTGGTCTTGAAGA-3′), mCXCL11 forward (5′-GACCAGGTTGGGCAAAGAGA-3′) and reverse (5′-GGCATCCTGGACCCACTTCT-3′), mGAPDH forward (5′-CAACTACATGGTCTACATGTTC-3′) and reverse (5′-CTCGCTCCTGGAAGATG-3′). The relative concentrations of each target template were calculated according to the comparative Ct method. The expressions of the target transcripts were standardized to the expression of GAPDH. RT-qPCR analyses were performed in triplicate.

### ELISA

For the *in vitro* experiments, irradiated tumor cells (5 or 10 Gy) and control cells were incubated in fresh medium for 24 h. For the *in vivo* experiments, tumors were harvested and placed in serum-free cold RPMI-1640 medium (1 mg of tissue per 10 ml of media) for 1 h, and then the tumor suspensions were centrifuged at 12,470 × g for 5 min. The medium and supernatants were collected and stored at −80°C. The levels of chemokines in the cell medium and tumor supernatants were quantified using Mouse CXCL9 ELISA Kit and Mouse CXCL11 ELISA Kit (Abcam, USA).

### Cytotoxic T-Lymphocyte Killing Assay

OT-I T cells were pre-activated with OVA peptide-pulsed spleen-derived DCs. MC38-OVA, MC38, EG7-OVA, or EL4 cells were subjected to 5 or 10 Gy of radiation (or sham-irradiation) and cultured in complete medium for 24 h, followed by labeling with 3 μM CFSE. The CFSE-labeled tumor cells were co-incubated at the indicated ratios with activated OT-I T cells for 4 h. After incubation, the cells were stained with 0.1 μg/ml DAPI for the flow cytometry assay. The percentage of specific cytolysis was defined according to the number of CFSE and DAPI double-positive cells.

### Combination Therapy of Established Tumors in Mice

Female C57BL/6 mice were injected subcutaneously with 0.5 × 10^6^ EG7-OVA or 2 × 10^6^ MC38-OVA tumor cells. The perpendicular tumor diameters were measured with a Vernier caliper every 2–3 days, and the tumor lengths were measured along two orthogonal axes (l and w) and calculated according to the equation tumor mean lengths = (l + w)/2. The tumors were randomly assigned by size to different treatment groups and treated with local irradiation. For local irradiation, mice were anesthetized by chloralic hydras injection. Established flank tumors (8–10 mm in length) were irradiated by X-rays generated from RS-2000 Biological Irradiator (RadSource, Canada) while the rest of the mouse body was shielded by lead shielding. The OT-1 T cells (5 × 10^6^ cells/mouse) were administered intraperitoneally the next day after irradiation. For the survival studies, mice were euthanized when the tumor lengths exceeded 15 mm.

### Statistical Analysis

Statistical analyses were performed using Prism 7 (GraphPad, Canada). All data were shown as the mean ± SD unless otherwise stated, and significant differences were determined using a two-tailed Student's *t*-test or ANOVA. *P* < 0.05 were considered statistically significant, ^*^P < 0.05; ^**^P < 0.01; ^***^P < 0.001. The results represent at least three experiments unless otherwise stated.

## Results

### Irradiation Increased the Release of TAAs From Tumor Cells

**Figure 1:**
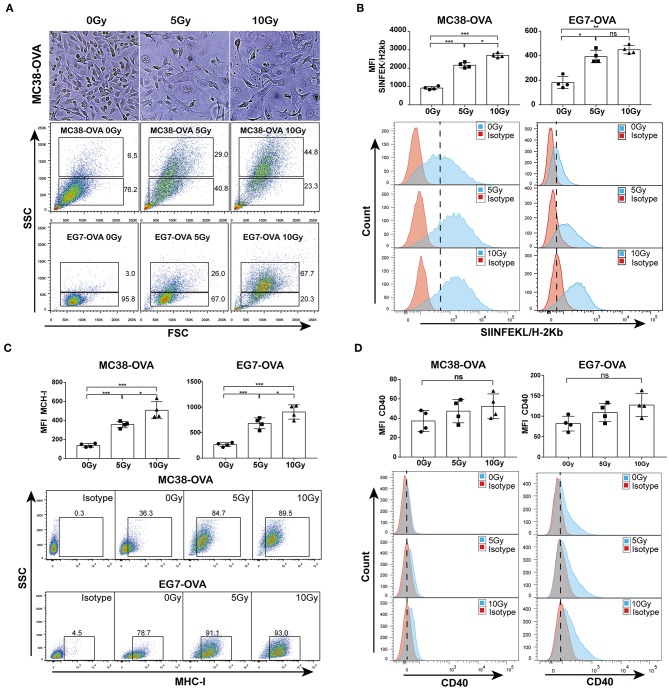
Irradiation increased the release of TAAs from tumor cells. **(A)** Microscopy images of MC38-OVA cells cultured for 48 h after irradiation (top panel); flow cytometry forward scatter and side scatter plots of irradiated or non-irradiated MC38-OVA and EG7-OVA cells cultured for 48 h (bottom panel). **(B)** The expression of the MHC class I-SIINFEKL complex on MC38-OVA and EG7-OVA cells cultured for 48 h after irradiation. **(C)** Flow plots and percentages of irradiated MC38-OVA and EG7-OVA cells cultured for 48 h and stained with isotype or MHC-I antibodies. **(D)** Histograms of irradiated EG7-OVA cells MC38-OVA and stained with CD40 antibodies. Representative results from one of at least three independent experiments are shown; ns, no significance; ^*^P < 0.05; ^**^P < 0.01; ^***^P < 0.001.

To study the TAAs released from irradiated tumor cells, OVA-expressing MC38-OVA colorectal carcinoma and EG7-OVA lymphoma cell lines were used in our study. Considerable morphological changes after irradiation in these cell lines were as expected. Light microscopy of cells cultured for 48 h after irradiation showed that the cell sizes were enlarged, and the nuclear-to-cytoplasmic ratio and number of prominent nucleoli were increased (Figure 1A, top). Flow cytometry confirmed the increased forward scatter and side scatter of the tumor cells following irradiation (Figure 1A, bottom). To evaluate whether irradiation could lead to an increase in TAA expression, an antibody against the complex of the MHC class I molecule (H-2Kb) bound to the ovalbumin peptide SIINFEKL was used in this study. We found a significant dose-dependent increase in the expression of the MHC class I-SIINFEKL complex on irradiated MC38-OVA and EG7-OVA cells compared with non-irradiated cells (Figure 1B). In addition, irradiation also increased the expression of the MHC class I molecule (H-2Kb) (Figure 1C). To exclude the effect of morphological changes on flow cytometry staining of TAAs and MHC-I molecules, CD40 was used as a positive and negative control for surface staining on EG7-OVA cells and MC38-OVA, respectively. There was no significant change in the expression level of CD40 on MC38-OVA cells, and a slight increase of CD40 expression was detected on EG7-OVA cells (Figure 1D). These results confirmed that irradiation could enhance the expression of TAAs and MHC-I molecules on the surfaces of tumor cells, which might be involved in antigen presentation.

### Irradiation Facilitated the Cross-Priming of Antigen-Specific T Lymphocytes *in vitro*

**Figure 2:**
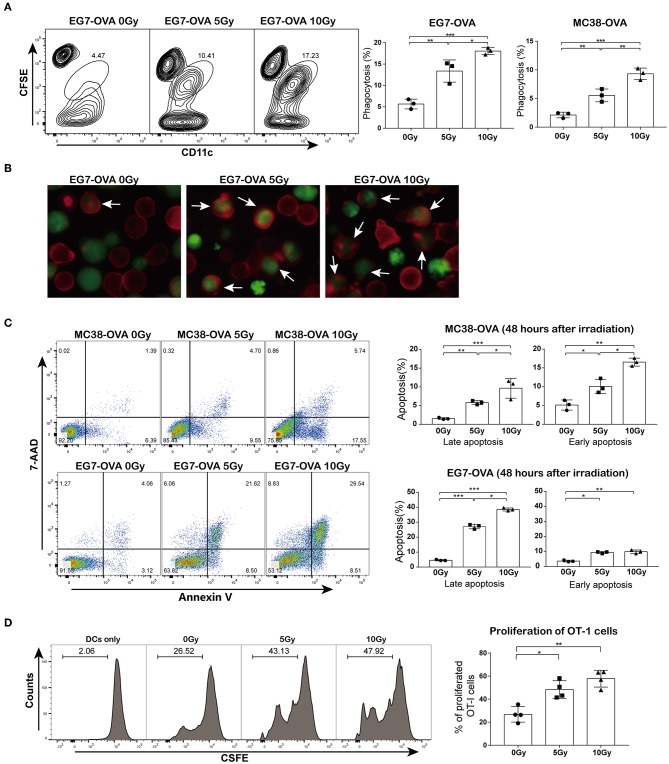
Irradiation facilitated cross-priming of OT-I T cells *in vitro*. **(A)** DC phagocytosis of tumor cells examined by flow cytometry. EG7-OVA and MC38-OVA cells were subjected to 5 or 10 Gy of radiation (or sham-irradiation) and then labeled with CFSE after 48 h of incubation in complete medium; DCs were stained with CD11c antibody and then co-cultured with irradiated tumor cells for 4 h. **(B)** An image of DC phagocytosis (arrows) in the presence of irradiated cells compared to that of non-irradiated cells (×200 magnification) obtained by fluorescence microscopy. **(C)** MC38-OVA and EG7-OVA cells received 5 or 10 Gy of radiation (or sham-irradiation), followed by staining with annexin V-PE and 7-AAD. The percentages of early and late apoptotic cells were determined and are shown in the right panel. **(D)** Irradiated or non-irradiated EG7-OVA cells were incubated with DCs. After 4 h of incubation, DCs were harvested and co-cultured with CFSE-labeled naïve OT-I T cells for 72 h. The proliferation of OT-I T cells was measured using the CFSE dilution by flow cytometry and quantified via calculation of the percentage of CFSE^+^ OT-I cells. Representative results from one of at least three independent experiments are shown. ^*^P < 0.05; ^**^P < 0.01; ^***^P < 0.001, by one-way ANOVA.

To explore whether irradiation could facilitate the cross-presentation and subsequent priming of antigen-specific T lymphocytes, we first assessed whether irradiated cells could be phagocytized by DCs more efficiently than non-irradiated cells. MC38-OVA and EG7-OVA cells were subjected to a single dose of 5 or 10 Gy of radiation (or sham-irradiation). The phagocytosis assay showed that the proportions of irradiated tumor cells bound and ingested by DCs were higher than those of non-irradiated cells (Figure 2A). Fluorescence microscopy confirmed an increase in irradiated cell uptake by DCs (Figure 2B). To investigate whether the increase in antigen uptake was correlated with apoptosis, both cell types were stained with annexin V and 7-AAD 48 h after irradiation. Indeed, irradiation induced both early and late apoptosis (Figure 2C).

Next, we evaluated whether the irradiation-enhanced phagocytosis of tumor cells by DCs was correlated with the priming of antigen-specific T lymphocytes. Cell division assay results showed that DCs pulsed with irradiated EG7-OVA cells significantly promoted OT-I T cell proliferation. However, OT-I T cells showed a slight proliferative response after co-culture with DCs pulsed with non-irradiated EG7-OVA cells (Figure 2D). Collectively, these findings suggested that irradiation augmented the cross-presentation and proliferation of antigen-specific T lymphocytes *in vitro*.

### Irradiation Enhanced Proliferation and Activation of Both Adoptively Transferred T Cells and Endogenous Antigen-Specific T-Lymphocytes *in vivo*

**Figure 3:**
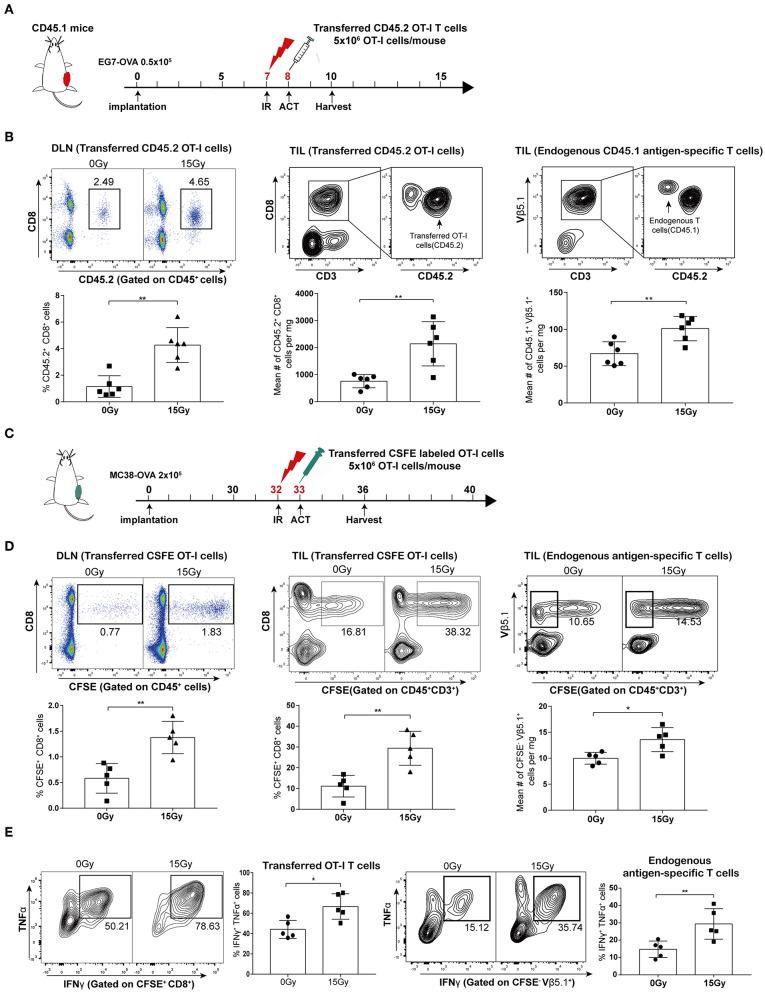
Irradiation enhanced the proliferation and activation of both adoptively transferred T cells and endogenous antigen-specific T lymphocytes *in vivo*. **(A)** Scheme of treatment: mice inoculated with EG7-OVA tumors received 15 Gy when the tumor lengths reached ~10 mm. OT-I T cells were transferred to mice the day after irradiation. **(B)** On day 4 after irradiation in the EG7-OVA model, the percentages of transferred CD45.2^+^ OT-I T cells in DLNs (left panel), and the absolute number of transferred CD45.2^+^OT-I T cells (middle panel), and endogenous CD45.1^+^TCRVβ5.1^+^ T cells (right panel) in tumor sites were analyzed. *n* = 6, one representative of three independent experiments. **(C)** Scheme of treatment in the MC38-OVA model. **(D)** On day 5 after irradiation, the percentages of transferred CFSE-labeled OT-I T cells (CFSE^+^ CD8^+^) in DLNs (left panel) and tumors (middle panel) were detected, and the absolute number of endogenous CFSE^−^TCRVβ5.1^+^ T cells (right panel) derived from tumors was calculated. *n* = 5, one representative of two independent experiments. **(E)** Expression of IFN-γ and TNF-α in transferred CFSE-labeled OT-I T cells (left panel) and endogenous CFSE^−^TCRVβ5.1^+^ T cells (right panel) on day 5 after irradiation in the MC38-OVA model. ^*^P < 0.05; ^**^P < 0.01; according to a two-tailed unpaired *t*-test.

To investigate the effect of irradiation combined with adoptive T cell therapy on T cell proliferation *in vivo*, we used transgenic OT-I/CD45.2/Rag^−/−^ mice as a source of CD8 T effector cells in conjunction with the EG7-OVA tumor model. We established EG7-OVA tumors on congenic mice carrying the Ly-5.1 (CD45.1) allele, and then these mice were locally irradiated with 15 Gy when the tumor lengths reached 10 mm. CD45.2 OT-I T cells were administered intraperitoneally into mice the day after irradiation (Figure 3A and Supplementary Figure S1). We assessed the proliferation of adoptively transferred CD45.2 OT-I T cells in DLNs and tumor tissues on day 4 after irradiation. Our data showed that irradiation significantly promoted the proliferation of the OT-I T cell population in both DLNs and tumors. Furthermore, cell number of the endogenous antigen-specific T cell population (CD45.1^+^ TCRVβ5.1^+^ cells) was also increased after irradiation (Figure 3B). Meanwhile, we established MC38-OVA tumors on C57BL/6 mice treated with irradiation, and then transferred CFSE-labeled OT-I T cells into mice the day after irradiation (Figure 3C). Similar to the results from the EG7-OVA tumor model, the proliferation of transferred (CFSE^+^CD8^+^) and endogenous (CFSE^−^ TCRVβ5.1^+^) T lymphocytes was increased after irradiation (Figure 3D). These results demonstrated that irradiation not only increased the expansion and migration of adoptively transferred CD8 T cells into tumor sites but also primed endogenous antigen-specific T-lymphocytes.

We further evaluated the effector function of the transferred OT-I T cells and endogenous antigen-specific T cells by performing intracellular staining for effector cytokines. As expected, we found increased expression of IFN-γ and TNF-α in T cells derived from irradiated tumors compared to that in T cells derived from non-irradiated tumors in the MC38-OVA model (Figure 3E). Altogether, these data indicated that tumor irradiation increased the activation and cytotoxicity of adoptively transferred T cells as well as endogenous antigen-specific T cells *in vivo*.

### Irradiation Facilitated Trafficking of Transferred OT-I T Cells Into the Tumor Tissues

**Figure 4:**
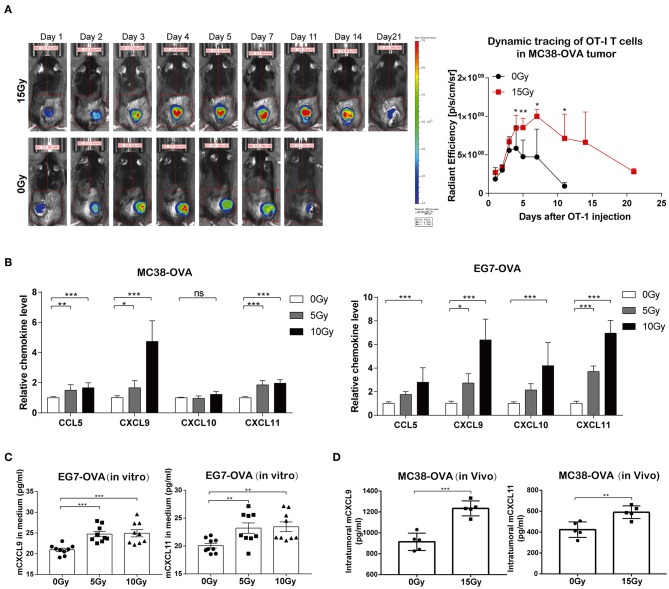
Irradiation facilitated the trafficking of transferred OT-I T cells into tumor sites. **(A)** Fluorescence signals from DiR-labeled OT-I T cells were visualized with an imaging system (left panel). OT-I T cells accumulated specifically in tumor tissues 24 h after the adoptive transfer of T cells, which peaked on day 4 and persisted up to day 21 in mice. Representative fluorescence signals from DiR-labeled cells relative to background fluorescence signals (right panel). Data are representative of two independent experiments (*n* = 4–6). **(B)** The expression of CCL5, CXCL9, CXCL10, and CXCL11 mRNA was analyzed by RT-qPCR in MC38-OVA and EG7-OVA cells 24 h after irradiation. Data are representative of at least three independent experiments. **(C)** Scatter graph displaying mCXCL9 and mCXCL11 (pg/ml) produced by EG7-OVA cells 24 h after irradiation *in vitro*. Data (mean ± SEM) are the combination of three independent experiments. **(D)** Scatter graph displaying mCXCL9 and mCXCL11 (pg/ml) produced by MC38-OVA tumor tissues 24 h after irradiation *in vivo*; *n* = 5, data are from one representative of two independent experiments; ns, no significance; ^*^P < 0.05; ^**^P < 0.01; ^***^P < 0.001.

T cell trafficking to tumor tissues following the adoptive transfer of T cells is important for the anti-tumor immune responses of ACT. We therefore explored the effect of irradiation on the ability of adoptively transferred OT-I T cells to traffic to tumors. C57BL/6 mice bearing MC38-OVA tumors were locally irradiated with 15 Gy when the tumor lengths reached ~10 mm. DiR-labeled OT-I T cells were administered intraperitoneally into mice the day after irradiation. FLI was performed at different time points over the next 3 weeks. The DiR fluorescence signals indicated that the transferred OT-I T cells accumulated specifically within tumors in both irradiated and non-irradiated mice 24 h after adoptive transfer. The peak signal intensity was obtained on day 4 after adoptive transfer and persisted up to day 11 in irradiated tumors, after which the signal intensity started to gradually diminish. The fluorescence signals from DiR-labeled cells persisted in irradiated tumors for up to 3 weeks. However, DiR fluorescence signals in non-irradiated tumors were weaker and cleared faster than those in irradiated tumors (Figure 4A and Supplementary Figure S2). These results implied that irradiation promoted the migration and persistence of adoptively transferred T cells *in vivo*.

We next sought to elucidate the mechanisms underlying the migration of adoptively transferred OT-I T cells to tumor tissues after irradiation. We found that the mRNA levels of CCL5, CXCL9, and CXCL11 were markedly increased in both MC38-OVA and EG7-OVA cells after irradiation *in vitro* (Figure 4B). We further examined the capacity of tumor cells to secrete CCL9 and CXCL11 in response to irradiation by performing *in vitro* and *in vivo* experiments. We found that the levels of CXCL9 and CXCL11 were significantly increased in both cell medium of irradiated EG7-OVA cells and the supernatants derived from MC38-OVA tumor tissues after irradiation (Figures 4C,D). Our findings implied that irradiation induced the release of CCL5, CXCL9, and CXCL11, which may produce a positive chemoattractant gradient in the tumor microenvironment and facilitate the migration of adoptively transferred T cells into tumor tissues.

### Irradiation Enhanced Tumor Cell Susceptibility to Cytotoxicity Induced by Antigen-Specific Cytotoxic T Lymphocytes

**Figure 5:**
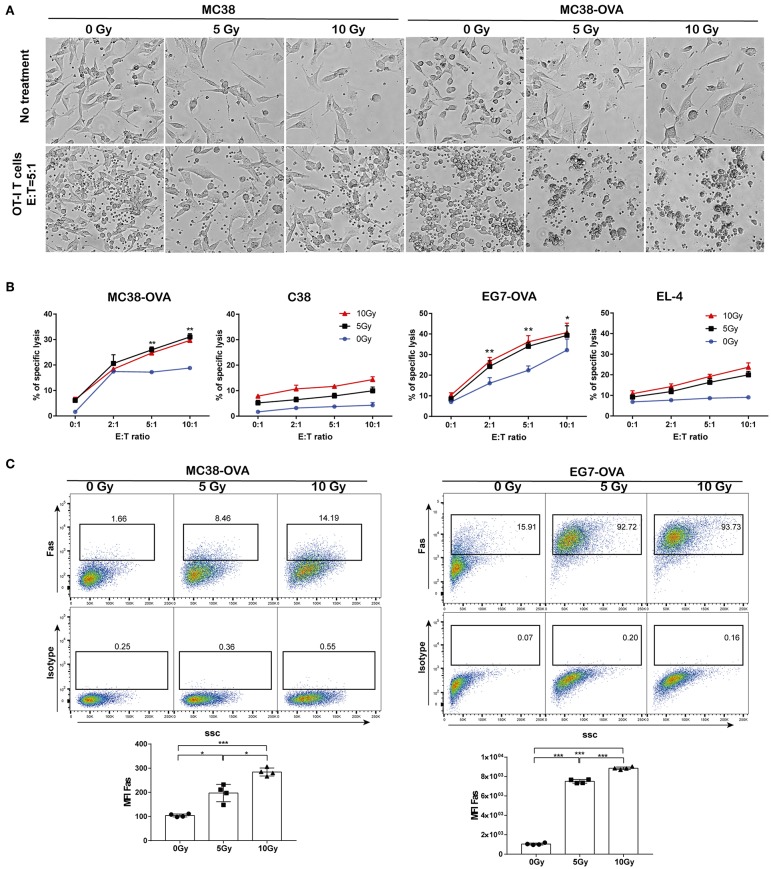
Irradiation increased tumor cell sensitivity to OT-I T cell-mediated lysis. MC38-OVA, MC38, EG7-OVA, or EL4 cells were subjected to 5 or 10 Gy of radiation (or sham-irradiation), and then rested in complete media for 24 h. **(A)** Microscopy images of MC38-OVA or MC38 cells co-incubated with activated OT-I T cells for 24 h. **(B)** CFSE-labeled MC38-OVA, MC38, EG7-OVA, or EL-4 cells were co-incubated with the indicated ratios of OT-I T cells for 4 h. These cells were harvested and stained with DAPI before analysis by flow cytometry. OT-I T cell-mediated lysis was defined by cells that were both CFSE and DAPI positive. **(C)** Fas expression on MC38-OVA (left panel) and EG7-OVA cells (right panel) was measured by flow cytometry 24 h after irradiation. Representative results from one of at least three independent experiments are shown. ^*^P < 0.05; ^**^P < 0.01; ^***^P < 0.001, by one-way ANOVA.

Next, we examined whether irradiation could improve the lytic effects on tumor cells of antigen-specific T lymphocytes. MC38-OVA and EG7-OVA cells received 5 or 10 Gy of radiation (or sham-irradiation), and then the cells were used as target cells for lysis by activated OT-I T cells. EL-4 and MC38 cells were used as the antigen-negative controls, respectively. Our results showed an increase in the lysis of MC38-OVA and EG7-OVA cells at the indicated E:T ratio (Figures 5A,B). Conversely, irradiation failed to increase the lysis of the MC38 and EL-4 cells at any E:T cell ratio. Our results suggested that irradiation sensitized MC38-OVA and EG7-OVA cells to the cytotoxicity mediated by antigen-specific T lymphocytes.

The Fas/Fas ligand system is a major pathway for the induction of apoptosis that contributes to the cytotoxic activity of CTLs (17). Hence, we examined the cell surface expression of Fas following irradiation, which may have impacted the lytic effects of CTLs. Our data showed that Fas expression on tumor cells was increased in a dose-dependent manner following irradiation (Figure 5C). These results implied that the upregulation of Fas expression on tumor cells by irradiation could improve their lytic susceptibility to antigen-specific T-lymphocytes, which may be one of potential mechanisms underlying the synergistic antitumor activity of adoptive T cell therapy and radiotherapy.

### Combination of Irradiation and Adoptive T Cell Therapy Inhibited Tumor Growth and Prolonged Survival in Murine Models

**Figure 6:**
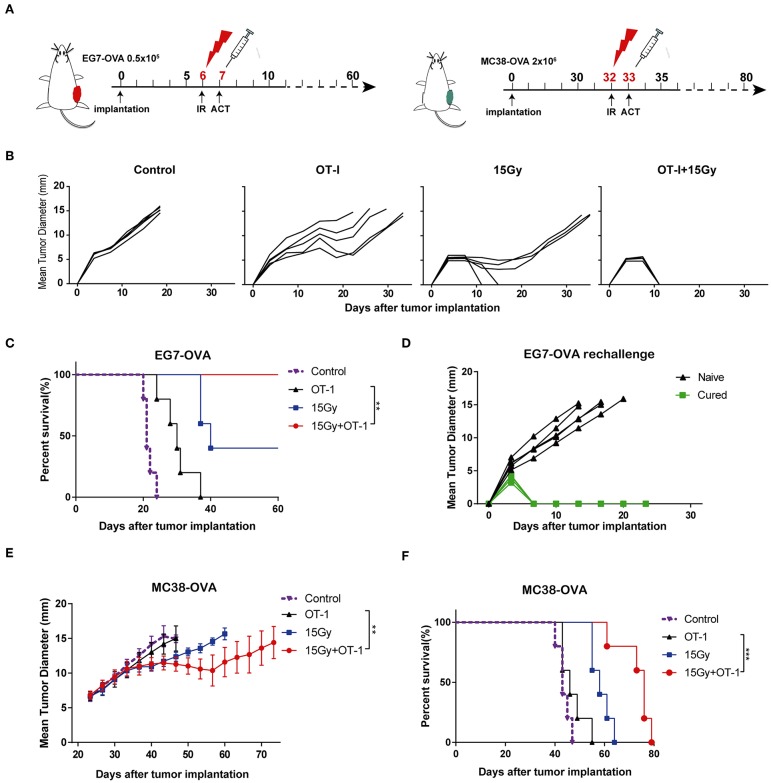
Local irradiation combined with adoptive T cell therapy inhibited tumor growth and prolonged survival in murine models. **(A)** Scheme of treatment. EG7-OVA or MC38-OVA tumors were inoculated into the flank of C57BL/6 mice. Tumors were subjected to 15 Gy or sham-irradiation when the mean tumor lengths reached ~8–10 mm, and the adoptive transfer of OT-I T cells was performed the day after irradiation. **(B)** The mean tumor lengths and **(C)** survival rates of mice in each group are shown (*n* = 5). **(D)** Mice that completely rejected the EG7-OVA tumors were re-challenged 2 months later by injecting 0.5 × 10^6^ EG7-OVA cells in the opposite flank (*n* = 5). **(E,F)** MC38-OVA tumors were inoculated in the flank of C57BL/6 mice by injecting 2 × 10^6^ cells. The treatment protocol was the same as that used for the EG7-OVA tumors. The mean tumor lengths and survival rates of mice in each group are shown (*n* = 5). All data are from one representative of two or three independent experiments. Percent survival of mice in the different groups depicted with a Kaplan–Meier plot, ^**^P < 0.01; ^***^P < 0.001.

We next investigated the effects of local tumor irradiation on the therapeutic efficacy of adoptive T cell therapy in murine models. C57BL/6 mice were inoculated subcutaneously in the flank with EG7-OVA tumors on day 0. Mice were locally irradiated with 15 Gy on day 6 when tumor lengths reached 8–10 mm and adoptively transferred OT-I T cells on day 7 (Figure 6A and Supplementary Figure S3). Perpendicular tumor diameters were measured every 2–3 days. As expected, adoptive OT-I T cells alone resulted in marginal tumor regression. However, the combination of adoptive T cell therapy and irradiation significantly inhibited tumor growth compared with that in other groups, and tumors were found to be completely regressed in all mice (Figure 6B). Additionally, the combination group showed significantly prolonged mouse survival compared with the other groups (Figure 6C). Furthermore, mice that completely rejected EG7-OVA tumors resisted subsequent re-challenge with EG7-OVA tumors in the opposite flank, demonstrating the presence of a protective antigen-specific memory response (Figure 6D). Similar to EG7-OVA tumors, established MC38-OVA tumors treated with irradiation and adoptive T cell therapy showed a significant growth delay (Figure 6E), and mice with these tumors showed prolonged survival (Figure 6F). These data demonstrated that local irradiation synergistically enhanced the therapeutic efficacy of subsequent adoptive T cell therapy, controlling tumor growth and improving the survival rates of mice.

## Discussion

CAR-T cell therapy, as part of a broader adoptive T cell therapy strategy, has emerged as an exciting new treatment option for hematological diseases. Nevertheless, adoptive T cell therapy in solid tumors remains a challenge, and spontaneous regression remains an exceptional phenomenon. Local tumor irradiation has been reported to enhance the antitumor efficacy of adoptive T cell therapy in mouse tumor models and preclinical research (18, 19). Irradiation can induce a multitude of alterations within the tumor microenvironment and systemic biological effectors outside the treatment field (20). In the present study, we focused on dissecting the immunological events associated with the irradiation of solid tumors in conjunction with adoptive T cell therapy.

Studies from several research groups have demonstrated that irradiation increased the expression of MHC class I molecules and TAAs in a dose-dependent manner in a variety of different tumor types (21, 22). MHC class I is responsible for the direct presentation of tumor antigen peptides to cytotoxic T lymphocytes (CTLs) via peptide–MHC complexes. By upregulating MHC expression, irradiation could prevent tumor cells from escaping immune recognition and elimination (23). In this study, we used tumor cell lines expressing model antigens to quantify the level of OVA antigen presentation in MC38-OVA colon adenocarcinoma and EG7-OVA lymphoma cells. We confirmed that irradiation enhanced the expression of OVA antigens present in MHC-I complexes on the cell surface in a dose-dependent manner. This raised the question of whether irradiation could potentially promote the engulfment of tumor cells by DCs to prime immune responses. DCs have the ability to present exogenous antigens on MHC class I molecules, which is called cross-presentation (24). During cross-presentation by DCs, exogenous antigens were internalized by phagocytosis, processed by proteasomes and presented on MHC class I molecules, which subsequently activated antigen-specific T lymphocytes (25). We demonstrated that the exposure of tumor cells to irradiation enhanced TAA uptake by DCs *in vitro*. Our results were in agreement with the findings of several previous studies, which showed that DCs could phagocytize irradiated tumor cells and induce a tumor-specific immune response (26, 27).

Increasing evidence from several studies emphasized the importance of TILs to the effects of cancer treatment in different tumor models (28–30). In this study, we found that tumor irradiation significantly enhanced the proliferation of antigen-specific T-lymphocytes *in vitro*. Furthermore, we showed that local tumor irradiation synergistically augmented the efficacy of adoptive T cell therapy *in vivo*. Our findings revealed that irradiation could not only promote infiltration and expansion of adoptively transferred T cells but also prime endogenous T responses against tumors. In addition, we observed that the activity of effector T cell was enhanced in adoptively transferred T cells and endogenous host T cells as measured by the expression of IFN-γ and TNF-α. Our results are consistent with previous reports that irradiation was associated with the increased expression of the effector cytokines IFN-γ and TNF-α by donor and host CD8 T cells (31). Thus, strategies that enhance antigen-specific T lymphocyte proliferation and infiltration into tumors could potentially result in significant anti-tumor benefits. Taken together, these observations indicate that the anti-tumor ability of irradiation combined with adoptive T cell therapy depends on antigen-specific T cell infiltration and functionality.

T cell trafficking into tumor tissues following adoptive T cell therapy is important to achieve successful anti-tumor immune responses for ACT (8, 32, 33). In this study, we observed that DiR-labeled OT-I T cells migrated into the tumor 24 h after adoptive T cell therapy, indicating that OT-I T cells could effectively traffic into tumors with excellent tumor-targeting capacity following intraperitoneal infusion. Our results demonstrated that irradiation facilitated OT-I T cell infiltration and persistence in tumor tissues compared with the control group. Numerous molecules, including T cell adhesion molecules and chemokines, have been shown to play critical roles in the engraftment of T cells to tumors (34, 35). However, it is still unclear which cell types are responsible for chemokine production, and the molecular mechanisms responsible for the upregulation of chemokines following irradiation are presently undefined. Dangaj et al. showed that tumor cells expressing CCL5 and macrophages and DCs expressing CXCL9 were critical for the migration of T cells into tumor tissues (35). In this study, we found that the expression of CCL5, CXCL9, and CXCL11 was increased in tumor cells after irradiation. Therefore, the increased release of chemokines in the tumor microenvironment induced by irradiation might enable the recruitment of activated T cells to tumor sites, thereby leading to a more effective T cell response.

Irradiation mediates multiple immunological effects against tumor cells, which renders tumors more susceptible to T cell-mediated antitumor effects (20). In addition to enhancing the release of tumor antigens, multiple other potential mechanisms exist by which irradiation can enhance irradiated tumor cell susceptibility. The enhancement of CTL lysis by the irradiation of tumor cells has been observed in several tumor models (36, 37). Consistent with previous reports, our results showed that irradiation sensitized MC38-OVA and EG7-OVA cells to the cytotoxic effects of OT-I T cells. The Fas/FasL pathway is one of the mechanisms used by cytotoxic T lymphocytes to directly kill specific target cells (36). Here, we found that dose-dependent increase in Fas expression was induced by irradiation in both MC38-OVA cells and EG7-OVA cells. Correspondingly, in established MC38-OVA and EG7-OVA tumor models, we found that local irradiation enhanced the antitumor effects of subsequent adoptive T cell therapy, resulting in synergistic effects on tumor regression and improved survival. These findings indicated that radiotherapy combined with subsequent adoptive T cell therapy may be a promising strategy for tumor treatment in the clinic.

## Conclusion

In summary, our study clearly supported the combination of irradiation and adoptive T cell therapy to improve local tumor control and survival in cancer therapies. The potential mechanisms underlying this synergism included the enhancement of the cross-priming, homing, and cytotoxicity of antigen-specific CD8 T cells by irradiation. In addition, T cell-attracting chemokines released from irradiated tumor cells could promote adoptively transferred T cell infiltration into tumors. These findings may provide a scientific basis for the further study of the combination of radiotherapy and immunotherapy in cancer treatment research.

## Data Availability Statement

All data generated or analyzed during this study are included in this published article. Further details are available from the corresponding author upon request.

## Ethics Statement

All procedures performed in studies involving animals were approved by the Fujian Medical University Institutional Animal Care and Use Committee (IACUC, No. 2017-031) in accordance with the ethical standards. All applicable international, national, and/or institutional guidelines for the care and use of animals were followed.

## Author Contributions

J-ZL and Q-YZ conceived and designed the study. J-ZL performed the experiments, analyzed the data, and drafted the manuscript. Y-YZ and MR cooperated in the establishment of tumor models. LC provided technical guidance. Q-YZ supervised the study and reviewed the manuscript. All authors read and approved the final manuscript.

### Conflict of Interest

The authors declare that the research was conducted in the absence of any commercial or financial relationships that could be construed as a potential conflict of interest.
