# Supplementary material for: Local Irradiation Sensitized Tumors to Adoptive T Cell Therapy via Enhancing the Cross-Priming, Homing, and Cytotoxicity of Antigen-Specific CD8 T Cells
Source: Front Immunol. 2019 Dec 11;10:2857. doi: 10.3389/fimmu.2019.02857 (PMC6919196; doi:10.3389/fimmu.2019.02857)
Supplement: Supplementary file 1 [file Data_Sheet_1.pdf]

Supplementary Material

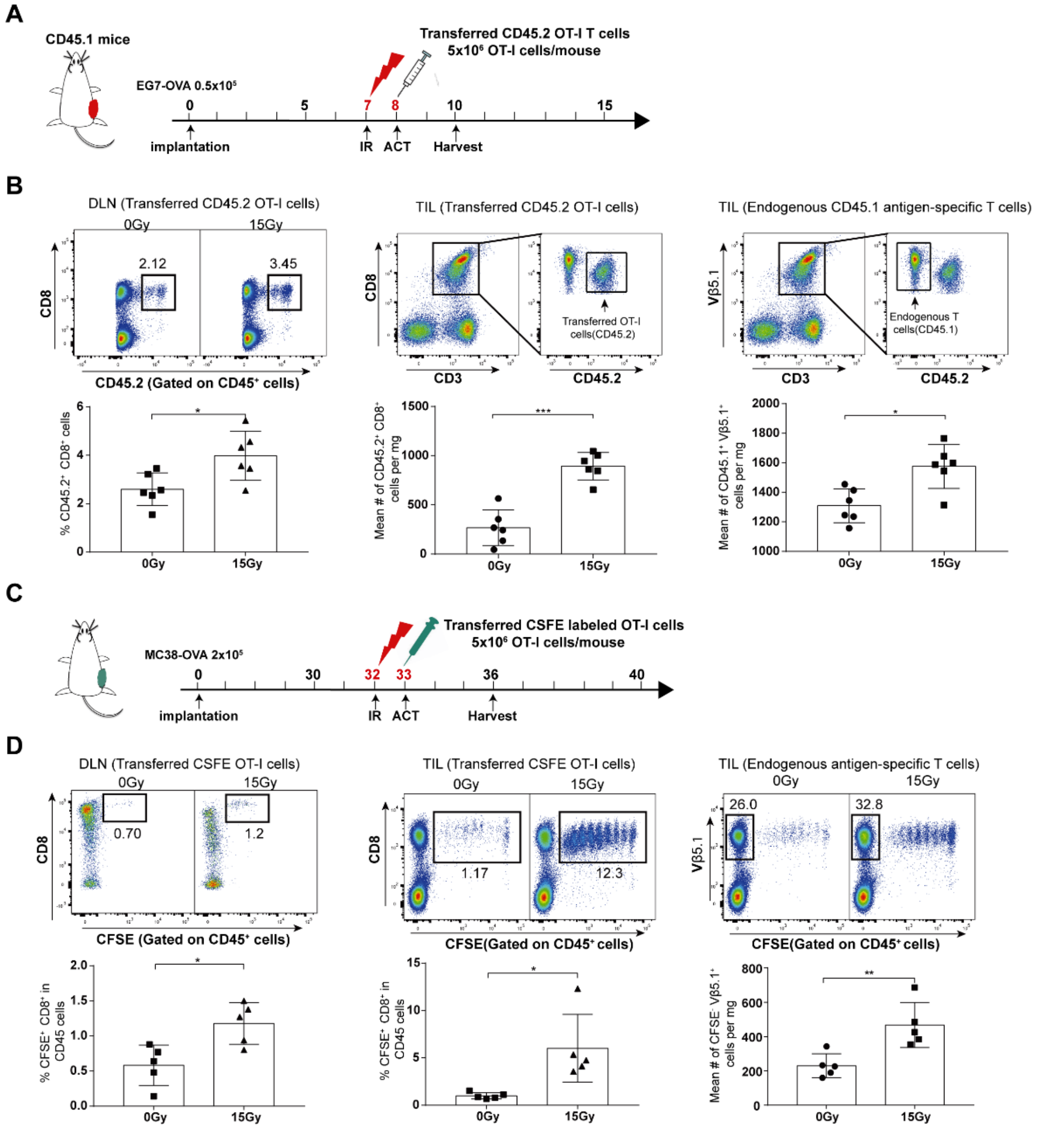

Supplementary Figure S1 | Mechanisms of the enhancement by local tumor irradiation of the anti-tumor effect of adoptive T-cell therapy for established tumors. **(A)** Scheme of treatment: mice inoculated with EG7-OVA tumors received 15 Gy of radiation or sham irradiation when the tumor lengths reached approximately 8-10 mm. OT-I T cells were transferred to mice the day after irradiation. **(B)** Dot plots of CD45.2^{+}OT-I T cells that were harvested from DLNs on day 4 after irradiation (left panel); absolute numbers of transferred CD45.2^{+}OT-I T cells (middle panel) and endogenous CD45.1^{+}Vβ5.1^{+} T cells (right panel), which were isolated from EG7-OVA tumors on day 4 after irradiation (n=6). **(C)** Scheme of treatment in the MC38-OVA model. **(D)** Percentages of transferred CFSE-labeled OT-I T cells (CFSE^{+}CD8^{+}) that were isolated from DLNs (left panel) and tumors (middle panel) on day 5 after irradiation, and the absolute number of transferred endogenous CD45.2^{-}Vβ5.1^{+} T cells (right panel) that were isolated from MC38-OVA tumors on day 5 after irradiation (n=5). **P* < 0.05; ***P* < 0.01; ****P* < 0.001, by a two-tailed unpaired t-test.

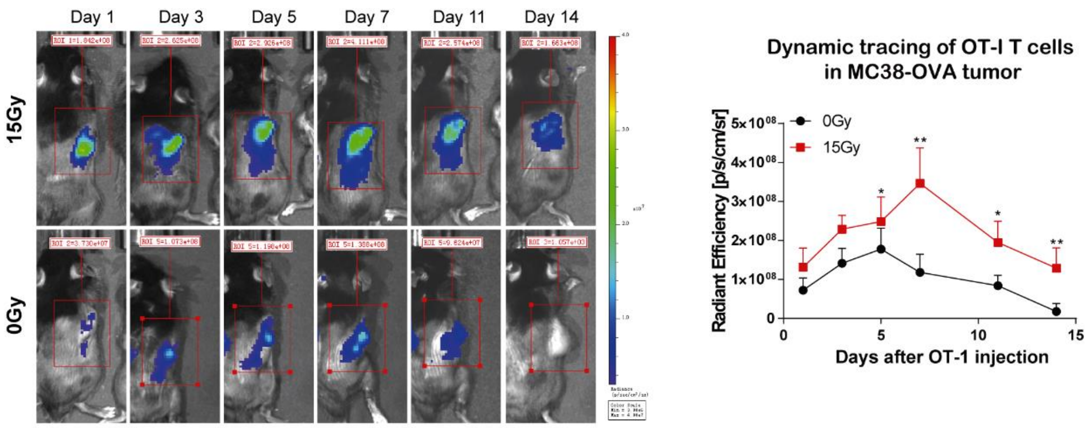

Supplementary Figure S2 | DiR-labeled OT-I T cells were visualized with an imaging system. OT-I T cells accumulated specifically in tumor tissues after the adoptive transfer of T cells into the MC38-OVA model. *$P < 0.05$; **$P < 0.01$; ***$P < 0.001$

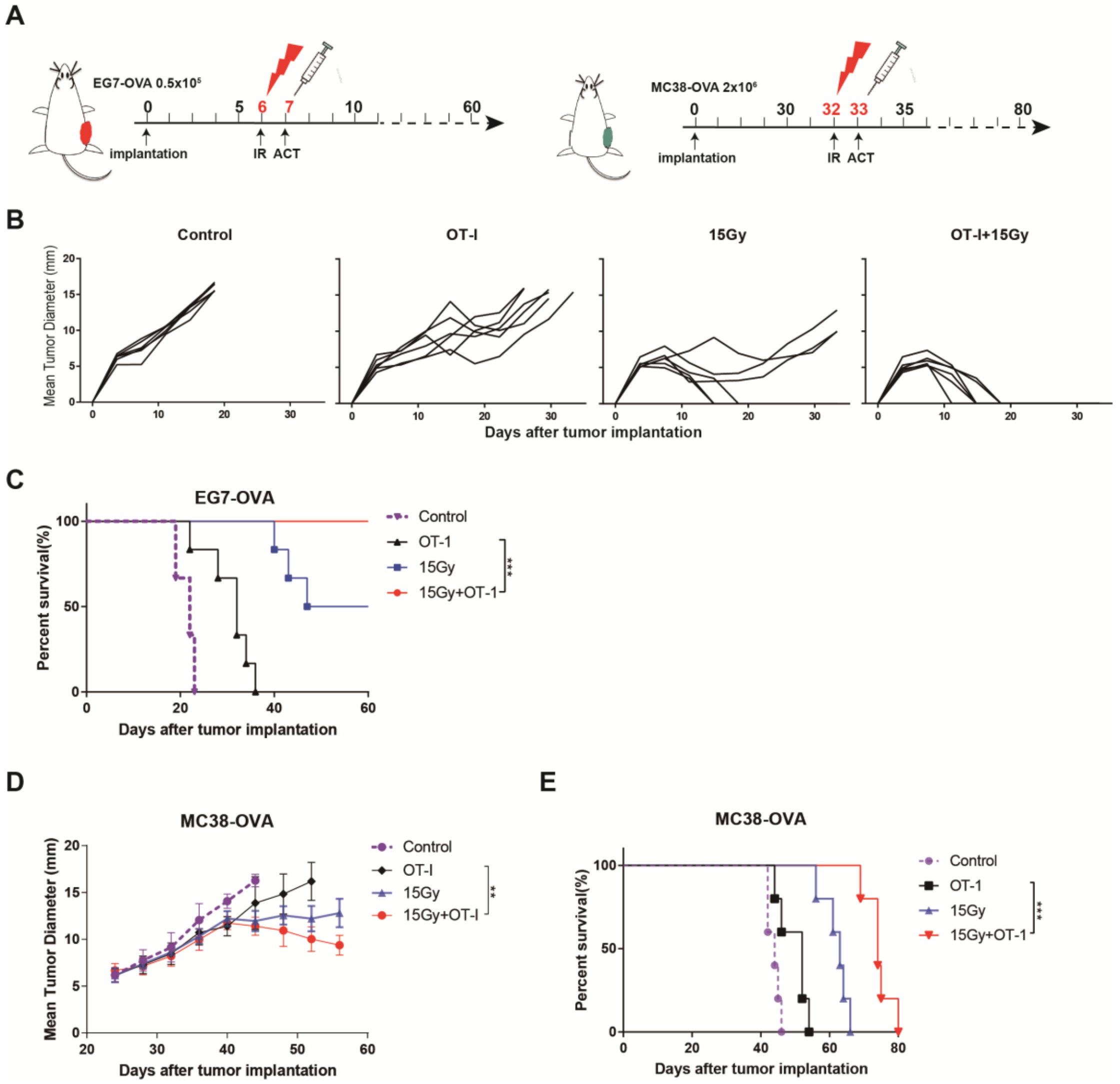

Supplementary Figure S3 | Local irradiation combined with adoptive T cell therapy inhibited tumor growth and prolonged survival in murine models. **(A)** Scheme of treatment. EG7-OVA or MC38-OVA tumors were inoculated into the flank of C57BL/6 mice. Tumors were subjected to 15 Gy or sham-irradiation when the mean tumor lengths reached approximately 8-10 mm, and the adoptive transfer of OT-I T cells was performed the day after irradiation. (**B**) The mean tumor lengths and (**C**) survival rates of the mice in each group are shown (n=5). (**D**-**E**) MC38-OVA tumors were inoculated in the flank of C57BL/6 mice by injecting 2×10^6 cells. The treatment protocol was the same as that used for the EG7-OVA tumors. The mean tumor lengths and survival rates of mice in each group are shown (n=5). All data are from one representative of two or three independent experiments. Percent survival of mice in the different groups depicted with a Kaplan-Meier plot, $^{**}P < 0.01$; $^{***}P < 0.001$.
